# Public Helping Reactions to Intimate Partner Violence against Women in European Countries: The Role of Gender-Related Individual and Macrosocial Factors

**DOI:** 10.3390/ijerph17176314

**Published:** 2020-08-30

**Authors:** Celia Serrano-Montilla, Inmaculada Valor-Segura, José-Luis Padilla, Luis Manuel Lozano

**Affiliations:** 1 Department of Methodology for Behavioral Sciences, University of Granada, 18011 Granada, Spain; celiaserrano@ugr.es (C.S.-M.); jpadilla@ugr.es (J.-L.P.); lmlozano@ugr.es (L.M.L.); 2 Department of Social Psychology, University of Granada, 18011 Granada, Spain

**Keywords:** intimate partner violence against women, helping reaction, gender-related factors

## Abstract

Public helping reactions are essential to reduce a victim’s secondary victimization in intimate partner violence against women (IPVAW) cases. Because gender-related characteristics have been linked widely to IPVAW prevalence, the study aimed to examine individual attitudes and perceptions toward different forms of violence against women, as well as gender-related macrosocial ideological and structural factors, in explaining helping reactions to IPVAW across 28 European countries. We performed multilevel logistic regression analysis, taking measures from the Eurobarometer 2016 (*N* = 7115) and the European Institute for Gender Equality datasets. Our study revealed a greater individual perceived IPVAW prevalence, positive perception about the appropriateness of a legal response to psychological and sexual violence against women partners, and less VAW-supportive attitudes predicted helping reactions (i.e., formal, informal), but not negative reactions to IPVAW. Moreover, individuals from European countries with a greater perceived IPVAW prevalence and gender equality preferred formal reactions to IPVAW. Otherwise, in the European countries with lesser perceived IPVAW prevalence and negative perceptions about the appropriate legal response to psychological and sexual violence, people were more likely to provide informal reactions to IPVAW. Our results showed the role of gender-related characteristics influenced real reactions toward known victim of IPVAW.

## 1. Introduction

Violence against women (VAW) is recognized as a violation of human rights and women’s freedoms and as a global obstacle to the achievement of equal power relations between men and women [1]. VAW has been widely defined as, “any act of gender-based violence that results in, or is likely to result in, physical, sexual or psychological harm or suffering to women, including threats of such acts, coercion or arbitrary deprivation of liberty, whether occurring in public or in private life” [1] (Art. 1, p. 2). In particular, intimate partner violence against women (IPVAW), a specific form of VAW by her partner or ex-partner, is one of the most frequent and is the focus of a substantial body of theoretical and empirical research [2,3,4]. To be a victim of IPVAW has been linked to aggravating impact not only on her physical, sexual, financial, reproductive, and psychosocial health but also on suicide behavior and her children’s well-being [2,5]. In this vein, 155 global governments recently established legal boundaries for IPVAW [6] and others, in addiction, prevention programs to combat this phenomenon [7]. Despite the awareness-raising context, a worrisome prevalence remains; overall, rates are approximately 35% across the world [8] and, in Europe, range from 13% in Spain, Austria, Croatia, Poland, and Slovenia to 32% in Latvia, Denmark, and Finland [9].

Empirical research has mainly focused on investigating IPVAW occurrence, while little scholar attention has been paid to other aspects that contribute to prevent this form of violence, such as public helping reactions [10]. Thus, within the ecological framework proposed by Heise [11], evidence have indicated that, beyond well-documented individual risk factors (e.g., age, marital status, and alcohol abuse) [12], gender-related macrosocial characteristics (i.e., ideological and structural) specific to gender inequality contexts—that is, societies where a power asymmetry between men and women is maintained—have been linked to physical, psychological, and/or sexual victimization by the current partner in the last 12 months [13,14,15,16,17]. Regarding ideological factors, for instance, higher IPVAW rates were found within countries that endorsed favored attitudes toward gender inequality, traditional gender roles, and the acceptability of IPVAW [3,13,17]. Likewise, according to structural indicators of women’s empowerment, societies where women held decreased life expectancy, literacy, economic resources, autonomy, and politic representation in comparison to men, showed higher prevailing IPVAW rates [3,18,19]. Extending the role of macrosocial factors toward helping reactions to IPVAW, we suggest that, in contexts where there is a tolerant or normalized climate toward IPVAW, the general population is less likely to report or react against such incidents [17,19,20,21]. Our work intends to contribute to a better understanding of society’s helping reaction to this problem, pointing toward whether individual and macrosocial gender-related factors facilitate or inhibit helping reactions to IPVAW across 28 European countries.

### 1.1. Gender-Related Factors and Helping Reactions to IPVAW

Helping reactions to IPVAW can be manifested in three types [21,22]: (a) *formal helping reactions* that include connections to formal resources and tangible/instrumental support (e.g., preferences for setting the law enforcement process in motion, health professionals, official services or organizations), (b) *informal helping reactions* that refer to a preference for personal intervention (e.g., emotional support to the victim, stopping the perpetrator, expressing disapproval), and (c) *negative helping reactions* that allude to a preference for nonintervention (e.g., ignoring, thinking that is not my business, avoidance of the victim or talking about IPVAW, and annoyance when the victim does not take the informal support or advice). Researchers have wondered what characteristics make individuals more prone to engage in different helping behaviors when they face IPVAW incidents. Traditionally, some coherent responses have been drawn from the bystander intervention model [23,24]. It suggests that prior to deciding to help, individuals move through several stages of the decision-making process: they must be aware of the IPVAW situation, interpret it is a problem, feel self-efficacy, take responsibility for intervening, think about what to do, have the willingness to intervene, and finally, translate such willingness into a real intervention. Different factors could affect different stages, facilitating or inhibiting the final help behavior [25]. Empirical data from this outlook have shown wide differences in helping behaviors according to individual factors [20,26]. Specifically, different studies have focused on determining the stages affected by gender-related intrapersonal characteristics –endorsing within societies where a power asymmetry between women and men are maintained– such as perceptions, beliefs, and general attitudes toward violence against women [20,27,28,29]. For example, victim-blaming attitudes, traditional gender role beliefs, hostile sexism, and acceptability of IPVAW have been linked to a lesser willingness to intervene in cases of IPVAW (i.e., a preference for calling law enforcement and a lack of preference for nonintervention) [22,27]. Moreover, the endorsement of myths or misconception beliefs about rape or IPVAW promotes a greater victim blame and exoneration of perpetrator, withholding the sense of personal responsibility to intervene [20,30,31]. Additionally, Beeble et al. [29] found, using a representative U.S. sample, IPVAW victims were more likely to be helped by those who had a consciousness about the high prevalence of IPVAW in their communities. Despite the well-documented role of above individual factors [32], Banyard’s [20,33] socio-ecological extension of the traditional bystander intervention model, in the “second action coil,” introduced distal setting characteristics from exosystem and macrosystem (i.e., the neighborhood, community, or country) where IPVAW took place to gain a deeper understanding of bystanders’ decisions to intervene in such incidences. Within the macrosystem, Heise [11] made a distinction between macrosocial characteristics, defining them as gender-related ideological or structural factors. Gender-related ideological factors allude to norms, beliefs, and values related to the status or roles of both men and women in the society. Otherwise, gender-related structural factors made reference to degree of real equality between men and women achieved in different areas (e.g., resources, political representation, work, education). Following Hofstede’s framework, dimensions of culture [34] have been used to understand variation in IPVAW across different countries [35,36,37,38]. Specially, masculine cultures (i.e., where “traditional” gender roles are strongly maintained in the culture; vs. feminine), greater power distance cultures (i.e., those where a power is not equally distributed among all members; vs. lower power distance) and individual cultures (i.e., the ties between individuals are loose, focusing on themselves and close family; vs. collective) show higher legitimization and rates of IPVAW [35,36,37,38,39]. However, to our knowledge, empirical evidence is still scarce in order to understand the differential role of gender-related ideological and structural macrosocial factors in helping reaction to IPVAW across European countries [40].

### 1.2. Current Research

Empirical evidence of helping reactions to IPVAW has been limited primarily to the examination of (a) bystander helping behaviors within high-risk situations of different gender-based violence [27,40,41]; (b) preferences toward intervention instead of real assisting reactions [22,42]; (c) small noncommunity samples (e.g., college students) [27,29]; and (d) risk factors at the individual level [21,33]. The current work extends the prior research by providing evidence about differences in real helping reactions to IPVAW across 28 European countries. To reach this aim, the objective of this research is twofold: (1) to examine the influence of European participants’ attitudes and perceptions toward different forms of VAW on the prediction of their formal, informal, or negative helping reactions to IPVAW, and (2) to analyze the macrosocial gender-related ideological and structural variables that can determine such reactions.

## 2. Materials and Methods 

### 2.1. Data Source 

Data were taken from Eurobarometer 85.3, a round of standard population-level public opinion surveys in Europe that are conducted by the European Commission [43]. This dataset is deposited in the GESIS (Leibniz Institute for the Social Sciences) repository; upon request, we obtained access to the surveys. In that round, Eurobarometer asked for different political and social aspects. We analyzed questions regarding perceptions, attitudes, awareness, legality, support, and the acceptability of different gender-based violence. From 4 June through 13 June, 2016—based on uniform methodological practices, such as a multistage random sampling design and a back translation of the target questionnaire to a different national language—a large community-based sample of the population from each EU member country was interviewed face-to-face (CAPI, Computer Assisted Personal Interview, was used, only when available). For the whole sample, EU countries were represented in proportion to their population sizes, ranging from 500 (Malta) to 1585 (Germany) with 27,919 participants aged 15 years and older. Data were de-identified and publicly available, thus no ethical approval was required. Details that are more technical could be checked on the Special Eurobarometer 449 report [43]. 

### 2.2. Study Population

According to the purposes of this study, we only selected participants that reacted to known women victims of intimate partner violence (i.e., within their circles of friends and family, immediate area, neighborhood, or where their worked or studied). Participants who knew men victims or both types, men and women victims were excluded. Therefore, a final analytic sample of 7115 participants from all 28 EU countries (ranging from 127 from Luxembourg to 432 from Sweden) was taken. Sociodemographic characteristics of the European population who knew a victim of IPVAW appear in Table 1.

### 2.3. Measures

We included in the analysis a set of gender-related variables for their conceptual links with helping reactions to IPVAW. Hence, perceptions and attitudes toward different forms of VAW were taken from Eurobarometer 85.3 for the individual level and macrosystem (hereafter country level) [43], and gender equality—for the country level—were taken from the European Institute for Gender Equality (EIGE) [44]. Likewise, covariate variables were introduced to ensure that the results of the current study could not be attributed to known confounding variables [24,45,46]. No conventional evidence about reliability of composite measures is provided (i.e., internal consistency) because quality of data of social surveys tend to be control *a priori* (e.g., sampling method, coverage, or measurement error).

#### 2.3.1. Outcomes

To measure helping reactions to IPVAW, we used an Eurobarometer multi-item survey question that asked for possible helping behaviors towards IPVAW victims in the past: (a) *formal helping reaction to IPVAW* formed by three items (i.e., speak to police, public or independent support services, and health professionals); (b) *informal helping reaction to IPVAW* measured by three items (i.e., speak to the people involved, a friend or a family member, and to another person or service); and (c) *negative helping reaction to IPVAW* measured by one item (i.e., no response when facing known cases of IPVAW). The stem of the multi-item survey question was “*Did you speak to anyone about this?*” with multiple possible answers by indicating which of the above helping behaviors they used when they knew a victim of IPVAW. Each item used a dichotomous answer format (0 = no mention, 1 = mention). Finally, formal, informal, and negative helping reactions to IPVAW were operationalized as at least the mention of one item. 

#### 2.3.2. Individual-Level Predictors: Gender-Related Attitudes and Perceptions

*Perceived prevalence of IPVAW.* This measure was shaped by one survey question asking respondents for how usual they thought IPVAW was in their own countries (“*In general, how common do you think that domestic violence against women is in [your country]?*”). It was a closed-ended question with four response options that ranged from 1 (very common) to 4 (not at all common). Higher scores meant a lesser perceived prevalence of IPVAW.

*Perceptions about the appropriateness of a legal response to psychological and sexual violence against partners.* The multi-item survey question was intended to obtain the respondents’ opinions about whether specific forms of psychological (e.g., “*repeatedly criticizing a partner to make them feel inferior*”) and sexual (e.g., “*forcing a partner to have sex*”) violence should be against the law. The response options for the three items ranged from 1 (wrong and already against the law) to 4 (not wrong and should not be against the law). Higher scores meant greater negative perceptions about the appropriateness of a legal response to psychological and sexual violence against partners.

*Violence against women (VAW)-supportive attitudes.* Data came from a multi-item survey question. It included four items on the level of tolerance, victim blaming, and the misconceptions toward violence against women (e.g., “*women often makeup or exaggerate claims of abuse or rape*, or *violence is often provoked by the victim*”). All items were Likert-type with four response options that ranged from 1 (totally agree) to 4 (totally disagree). Higher scores meant lesser VAW-supportive attitudes.

#### 2.3.3. Country-Level Predictors: Gender-Related Macrosocial Structural and Ideological Variables

Regarding structural variables, we used the *Gender-Equality Index* (GEI) from the European Institute for Gender Equality (EIGE) to examine the influence of gender equality at the country level. This index was operationalized based on six relevant domains [44]: (a) *work* (e.g., full-time equivalent rate, duration of working life, type of contract, and the prospects for career advancement); (b) *money* (e.g., mean monthly earnings and risk of poverty rate); (c) *knowledge* (e.g., people participating in formal or nonformal education, tertiary students in education, health and welfare, and humanities and art); (d) *time* (e.g., people doing the cooking and/or housework every day, and workers involved in voluntary or charitable activities at least once a month); (e) *power* (e.g., the share of members of parliament, board members of a central bank, and board members of research funding organizations), and (f) *health* (e.g., life expectancy at birth, people doing physical activities and/or consuming fruits and vegetables, and populations without unmet needs for medical examination). Index scores relied on gender gaps (i.e., the difference between women and men on different indicators that shape a domain), ranging from 1 (the lowest level of equality between females and males) to 100 (the highest level of equality between females and males). In our study, index values provided a measure of how close each EU member state was from achieving full gender equality in general and within specific domains in 2015 (for a further description of the index, see https://eige.europa.eu/gender-equality-index). Ideological variables were calculated aggregating individual scores of perceived prevalence of IPVAW, perceptions about the appropriateness of a legal response to psychological and sexual violence against partners and VAW-supportive attitudes by country. 

#### 2.3.4. Covariate Variables

*Sociodemographic variables.* Data for gender, age, marital status, working status, and years of education were included in the analysis. Excluding age, all categorical variables were converted to dummy variables. The reference group for each was male, unmarried, self-employed, and up to 15 years of education. The description of categorical variables is shown in Table 1.

*Response bias.* As in previous studies [46], two indicators of potentially biased responses were controlled: (a) interview privacy—measured by the number of people during the interview—was rated on a scale ranging from 1 (interviewer and respondent) to 4 (five or more people; 16.0% of Eurobarometer interviews were not conducted in private); and (b) respondent’s apparent interest during the interview—measured by respondent cooperation rated by the interviewers—ranged from 1 (excellent) to 4 (bad). Scores were reversed, such that higher scores meant more respondent cooperation (M = 3.60; SD = 0.63). 

### 2.4. Data Analysis 

First, we analyzed missing data; as we found a small number of these data (only 0.9% for marital status and 4.6% perceptions about the appropriateness of a legal response to psychological and sexual violence against partners), no imputation method was used [47,48]. Second, we calculated the descriptive statistics for the analyzed variables. Third, we assumed that the responses of participants (Level 1) from the same European country could be correlated because of sharing the same (or very similar) contexts (Level 2). With that in mind, we estimated a set of multilevel logistic regression models to account for participants’ formal, informal, and negative helping reactions to IPVAW [49]. These models allow the inclusion of additional error terms to reflect the intricate pattern of variation due to the hierarchical structure of the data [50]. Continuous variables were mean centered. Level 1 was centered using the mean of each country (group-mean centered), and the mean of all the countries (grand-mean centered) was used at Level 2. Then, we ran Model 0, an intercept-only model with random intercepts and without predictors, to determine how much variance of participants’ formal, informal, and negative helping reactions to IPVAW is explained by between-countries variation. After that, we performed a set of random-intercept and fixed-slope models with different gender-related predictors. Specifically, Model 1 introduced sociodemographic variables to control such variables in the following models. Model 2 included the attitudes and perceptions toward various forms of VAW at the individual level, and Model 3 added the previous ones at the country level (i.e., gender-related macrosocial ideological variables). Aggregating variables from Level 1 into grouping variables at Level 2 (e.g., countries) is a common practice in multilevel modeling to take care of the ecological fallacy when testing micro- and macro-level relationships [51,52]. Model 4 is the final model, where we additionally tested the effect of gender-related macrosocial structural predictor at the country level (i.e., objective gender equality). Therefore, these models tested the effect of macrosocial variables on participants’ helping reactions to IPVAW beyond the impact of previous individual covariates of the study. Likewise, Model 4 was reanalyzed with the two indicators of potentially biased responses (i.e., interview privacy and respondent’s apparent interest during the interview) to ascertain if biased responses conditioned results obtained in Model 4. No multicollinearity problems emerged (VIF < 5.0) [53] for any model. We used the Laplace approximation as estimation method. To compare the different model fits, we estimated the deviance, intra-class correlation (ICC), Akaike information criterion (AIC), and Bayesian information criterion (BIC). All analyses were performed with the lme4 package (version 1.1-21) implemented in R statistical software (version 3.5.3, R Core Team, Auckland, New Zealand) [54].

## 3. Results

The descriptive results of the key predictors at the individual and country levels are displayed in Table 2. Before analyzing the target models, we ran the intercept-only model to test the influence of Level 2 (i.e., countries) on the variability of helping reactions to IPVAW (see Model 0 in Supplementary Materials Table S1). ICC suggested small to medium effects for outcome variables. A total of 2%, 5.3%, and 5.2% of the variance in formal, informal, and negative helping reactions to IPVAW, respectively, were attributed to differences between countries. Although the effects were moderate, we took a multilevel approach by considering that a small or medium ICC index could detect substantial Level 2 relationships, which were not evident in Level 1 [55,56,57].

### 3.1. Individual Sociodemographic and Attitudinal Characteristics Influence on Formal, Informal, and Negative Helping Reaction to IPVAW

Table 3 shows the results from random-intercept and fixed-slope multilevel logistic regression models predicting helping reactions to IPVAW from individual characteristics (i.e., sociodemographic variables and perceptions and attitudes toward different forms of VAW). 

When gender-related perceptions and attitudes were introduced, after controlling for sociodemographic variables, the lower values of deviance, AIC, and BIC in Model 2 indicated a better fit to data in comparison to Model 1. Thus, results indicated that perceived prevalence of IPVAW, perceptions about the appropriateness of a legal response to psychological and sexual violence against partner, and VAW-supportive attitudes significantly contributed to the prediction of helping reactions to IPVAW. In particular, the odds of formal helping reactions to IPVAW increased when individuals showed a higher perceived prevalence of IPVAW (OR = 0.70; 95% CI: 0.62–0.80; *p* < 0.001) and a positive perception about the appropriateness of a legal response to psychological and sexual violence against partners (OR = 0.77; 95% CI: 0.67–0.90; *p* < 0.001). Informal helping reactions to IPVAW were predicted by a greater perceived prevalence of IPVAW (OR = 0.89; 95% CI: 0.81–0.98; *p* = 0.01) and lesser VAW-supportive attitudes (OR = 1.32; 95% CI: 1.19–1.46; *p* < 0.001). Finally, individuals with a greater perceived prevalence of IPVAW (OR = 1.20; 95% CI: 1.10–1.32; *p* < 0.001), with nonsupportive attitudes toward VAW (OR = 0.73; 95% CI: 0.66–0.81; *p* < 0.001), and positive perception about the appropriateness of a legal response to psychological and sexual violence against partners (OR = 1.14; 95% CI: 1.01–1.28; *p* = 0.03) were less likely to show negative helping reactions to IPVAW. Perception about the appropriateness of a legal response to psychological and sexual violence against partners did not explain informal helping reactions (OR = 1.00; 95% CI: 0.98–1.02; *p* = 0.88), and VAW-supportive attitudes did not explain formal helping reactions (OR = 1.14; 95% CI: 1.00–1.31; *p* = 0.06).

### 3.2. Gender-Related Macrosocial Ideological and Structural Factors as Country Predictors of Formal, Informal, and Negative Helping Reaction to IPVAW 

Table 4 illustrates the results from random-intercept and fixed-slope multilevel logistic regression models predicting helping reactions to IPVAW after adding gender-related ideological (Model 3) and structural (Model 4) characteristics at the country level. As Model 3 shows, there was a slightly better fit of data (i.e., AIC, BIC, and deviance indices) after introducing gender-related macrosocial ideological characteristics at the country level. The effects of respondents’ individual variables remained similar, but a country’s perceived prevalence of IPVAW, perceptions about the appropriateness of a legal response to psychological and sexual violence against partners, and VAW-supportive attitudes explained an additional proportion of the random variance in helping reactions to IPVAW.

This fact was indicated by the reduction in the country-level variance from Model 2 to Model 3 (i.e., between 0.04 and 0.09 for three outcome variables). Unlike the individual gender-related characteristics, results showed that formal helping reactions were not linked to any macrosocial ideological predictor at country level. Regarding informal helping reactions to IPVAW, countries with a lower adherence to VAW-supportive attitudes had greater odds of choosing informal helping reactions to IPVAW (OR = 3.23; 95% CI: 1.58–6.63; *p* < 0.01). Conversely to the results at the individual level, perceptions about the appropriateness of a legal response to psychological and sexual violence against partners had a significant effect at the country level (OR = 6.78; 95% CI: 2.00–23.0; *p* < 0.01); the odds of informal helping reactions to IPVAW increased in countries where there was a negative perception about the appropriateness of a legal response to psychological and sexual violence against partners. In addition, European countries with a lesser perceived prevalence of IPVAW significantly predicted greater odds of informal helping reactions (OR = 2.25; 95% CI: 1.14–4.44; *p* = 0.02). Similarly, negative helping reactions to IPVAW had two significant macrosocial ideological predictors at the country level: perceptions about the appropriateness of a legal response to psychological and sexual violence against partners (OR = 0.20; 95% CI: 0.06–0.71; *p* = 0.01) and VAW-supportive attitudes (OR = 0.31; 95% CI: 0.15–0.64; *p* < 0.01). Thus, European countries that endorsed lesser VAW-supportive attitudes were less likely to react to IPVAW negatively. Unexpectedly, a lesser likelihood of a negative helping reaction to IPVAW was found within countries with higher negative perceptions about the appropriateness of a legal response to different behaviors related to sexual and psychological violence against intimate partners.

Finally, we found that the inclusion of gender-related macrosocial structural characteristics at the country level (Model 4) did not significantly improve the model fit. However, a relevant finding was found regarding formal reactions to IPVAW. In particular, countries with greater objective equality between women and men (OR = 1.03; 95% CI: 1.02–1.06; *p* < 0.001) were more likely to have formal helping reactions to IPVAW, but no associations were found regarding informal and negative reactions to IPVAW. Moreover, a lower VAW-supportive attitude at individual level (OR = 1.15; 95% CI: 1.001–1.32; *p* = 0.047) and a greater perceived prevalence of IPVAW at the country level (OR = 0.46; 95% CI: 0.28–0.76; *p* = 0.002) gained a significant role, predicting formal helping reactions to IPVAW. 

### 3.3. Response Bias 

After controlling for response bias in Model 4 (i.e., interview privacy and a respondent’s apparent interest during the interview variables), the results were similar (see Supplementary Materials Table S1 to compare models). Thus, the results yielded a lack of response bias because estimates were not affected by the number of people during the interview or by the respondent’s apparent interest during the interview. However, the number of people during the interview had a positive and significant effect on the odds of formal helping reactions to IPVAW (OR = 1.21; 95% CI: 1.04–1.40; *p* = 0.01).

## 4. Discussion

Although there is well-known evidence concerning the barriers that IPVAW victims face when weighing the decision to disclose the violence they have experienced [58], the psychosocial characteristics—at different levels of social ecology—that affect public decisions to provide help remain unclear [33]. To clarify this gap, we analyzed differences in individuals’ helping reactions to known IPVAW victims across 28 European countries by examining whether (a) at the individual level, gender-related attitudes and perceptions toward different forms of VAW would replicate prior effects with real self-reported help, and (b) at the country level, gender-related macrosocial ideological and structural characteristics would promote helping reactions to known IPVAW.

Regarding the first aim, we found that European individuals who perceived a greater prevalence of IPVAW in their own countries and endorsed lesser VAW-supportive attitudes (i.e., tolerance, victim-blaming, and misconceptions toward VAW) were more likely to provide formal (i.e., talk with police, public services, or health professionals) and informal help (i.e., talk to the people involved, a friend or a family member, or another person or service) to the known victim. In addition, they did not show negative helping reactions to IPVAW (i.e., to avoid speaking with anyone). In the case of perceptions about the appropriateness of a legal response to psychological and sexual violence against partners, our results did not yield an effect on informal helping reactions to IPVAW, but European individuals who perceived psychological and sexual violence as wrong and already against the law were more likely to react formally toward a known victim of IPVAW and were less likely to react in a negative way [22,29,59,60]. Importantly, these effects remained significant even after controlling for several sociodemographic potential confounding variables known to be associated with bystander intervention in different forms of VAW [20,24,45]. Our findings confirm the well-known evidence based on the bystander intervention model [23], underlying that people who think IPVAW is common in a community—which may be a potential marker of perceived severity—were more likely to report it positively [61]. Besides, in line with prior research, unprejudiced attitudes and perceptions toward different forms of violence against women also influence bystanders’ decisions to help IPVAW victims [22,27]. Thus, during the decision-making process to intervene, these variables would be key points, helping to interpret the situation as unequivocally requiring an intervention, and in turn, translating this intention into an actual behavior.

We should interpret country-level findings cautiously because scarce variance was contributed by macrosocial factors, however we do think the more worthy and novel results are those derived from the analyses of gender-related ideological and structural factors (aim 2). Based on Banyard’s proposal [33], our results support and extend prior evidence about the importance of the culture –that is, particular values, practices, norms, and ideological beliefs established on social environment and adhered to by a majority of citizens [62]–that make up the process of deciding to provide help toward known IPVAW victims occurs [20,27,33]. 

First, in line with results at the individual level, people from countries that endorsed lower VAW-supportive attitudes (i.e., intolerance, lack of victim blaming, and suitable conceptions of different forms of VAW) were more prone to engage in informal helping reactions to IPVAW, as well as less likely to react negatively. It seems to note the relevance of social and cultural norms (i.e., gender-related ideological characteristics) established in the societies or communities where people live, defining the positions of women as inside or outside of traditional patriarchal stereotypes [28]. When society’s shared norms and ideological beliefs do not emphasize traditional ideas, such as victims provoking the violence or causing their own troubles, IPVAW is acceptable in all or some circumstances [20,24,63,64], some behaviors specific to IPVAW are not considered wrong [65,66], or this problem is not widespread [67], people feel accountability to engage in actions against IPVAW. Probably, they consider such violence as a threat to personal or community well-being and make the decision to help [67,68]. Likewise, according to Hofstede, Hofstede, and Minkov [34], the above tendencies could be also specific to European feminine countries since those uphold gender roles overlap (i.e., men and women are believed to be modest, caring, and concerned with the quality life) in comparison to masculine countries which emphasize on the differences established by traditional gender roles (e.g., men are considered to be dominant, competitive, and focused on material success, whereas women are assumed to be modest, caring, cooperative, and concerned with the quality of life) [34]. Thus, literature indicated that people from countries which endorsed less traditional gender roles (i.e., feminine countries), were more unlikely to legitimate IPVAW (i.e., blame and excuse violence) in comparison to those countries where people maintained greater traditional gender roles (i.e., masculine countries) [37,69], being this fact related to willingness to help IPVAW victims.

Second, in countries with a perception of a greater IPVAW prevalence, individuals were more disposed to engage in formal helping reactions compared to those in countries with less perceived IPVAW prevalence. Conversely to the individual-level findings, countries that endorsed lesser perceptions of IPVAW prevalence (vs. those with higher perceptions of IPVAW prevalence) showed greater individual informal helping reactions to IPVAW. Likewise, in European societies that maintained negative perceptions about the appropriateness of a legal response to sexual and psychological violence (i.e., it is not wrong and should not be against the law), individuals were more likely to provide informal helping reactions to known IPVAW victims and were less likely to help victims negatively (no relation was found with formal helping reactions to IPVAW). Currently, VAW is no longer officially considered a private matter within European Union countries. National laws penalize it, and primary prevention initiatives have focused on spreading messages to communities to increase visibility about all forms of this public and social phenomenon [6,7,33]. To some extent, this could explain our results because, in general, an aware environment could make feel people obliged to provide some type of help regardless of the prevailing gender-related ideological characteristics toward VAW within the country. Indeed, we checked that informal resources were more preferred, even though IPVAW was considered uncommon and sexual and psychological violence were not perceived as harmful. Some explanations emerge from this fact, suggesting other factors could influence on the associations. Thus, informal helping reactions might be chosen when the incident is perceived as less severe [60,70]. Even today, society continues to relate IPVAW mainly with physical violence, without considering the damage of other forms, such as sexual (e.g., forcing a partner to have sex) or psychological violence (e.g., control over a partner or yelling) [71,72]. Likewise, prior research indicated a smaller “bystander effect”—that is, intervention is less likely when more individuals are present—is found when victims belong to in-group or victims were known, such as our targeted population [63,70,73]. Otherwise, literature points toward countries with a more collectivist orientation (vs. individualist) [34] people tended to avoid going to formal resources (e.g., justice system) because they delegitimize such helping sources for conflicts involving primary social relationships [74]. Despite that, our findings seem to note an essential point: the type of help changed according to gender-related macrosocial ideological characteristics. While formal helping reactions to IPVAW are chosen by European countries with progressive perceptions and attitudes toward different forms of VAW, informal helping reaction were preferred in European countries without such progressive views. Further research should clarify why people prefer to intervene in informal ways (vs. formal ways) by examining the interplay between gender-related ideological characteristics and awareness about measures against IPVAW, the characteristics of victims (i.e., stranger or known victim), the severity of such violence, and the country’s degree of collectivism (vs. individualism) on helping reactions to IPVAW across European countries. 

Third, results suggested that countries with higher gender equality (i.e., equality between women and men in quality of knowledge, work, money, power, health, and time) were more likely to provide formal help, but no effects on informal and negative helping reactions to IPVAW were found. Substantial empirical data support the notion that IPVAW prevalence [3,13,39], as well as the justification of IPVAW [46,75], is significantly rooted in a gender inequality or larger power distance societies [34], however to our knowledge, we provide the first support linking gender-related macrosocial structural gender equality and the formal reaction to IPVAW within the European context. This result is particularly important because it reflects that living in a society that promotes and, indeed, consolidates gender equality by means of different domains (e.g., economy, political representation, and health status) models people’s helping attitudes and behaviors, specifically, those officially promoted by the government [76]. 

Our empirical findings also provide practical implications for the prevention of IPVAW. Recent evidence suggests that prevention initiatives should focus on transforming power inequality between men and women across different levels of social ecology [7,11,77]. We found marked individual and macrosocial factors. Within the European context, our work highlights that efforts should be mobilized for structural or objective indicators related to gender inequality, as well as perceptions and attitudes toward different forms of VAW (e.g., psychological and sexual violence). Thus, witnesses may move toward active participants who reject and interrupt violence and foster recognition that everyone has to be part of the solution [78]. According to the core principles for effective programming to prevent violence against women and girls [77], our results support the implementation and development of primary prevention across two spheres, thereby encouraging personal and collective critical thought [77]. First, at the country level, the European governments should generate strong national legislations against IPVAW, and they should give responsibility to women’s rights organizations in the local area to lead changes of customary norms or laws, in which negative perceptions and attitudes toward VAW are sustained and undertaken [79]. Second, at the individual level, aspirational programming has been supported. It focuses on working on the key issues by presenting specific real-world examples where people are involved [80,81,82]. In this vein, aware individuals will be potential agents of change (e.g., within group conversations) [77,83]. In short, multiple agents across multiple levels have the power to change social norms (i.e., focusing on key risk factors as perceptions and attitudes toward IPVAW) and to encourage personal and collective critical thoughts about violence against women. 

Finally, some limitations challenge the validity and generalizability of our findings. First, Eurobarometer survey questions are intended to measure public opinions about a broad range of topics in a big number of countries. Data quality depends on differences in administration modes, translations errors, sampling methods, and so forth, as in any international survey project. The several quality controls the Eurobarometer core team performs made us feel confident about data quality. Nevertheless, further research should examine equivalence to improve the validity of additional international comparisons [84,85]. Second, the explained variance at the country level is from fair to medium, and we should interpret the results with caution, paying more attention to those derived from individual level. Third, the reference period was not the same for the entire sample. In particular, structural indicators related to gender inequality were taken from a year before. Fourth, the cross-sectional design of our study prevents the possibility of establishing any causal connection among the associations found. In the future, longitudinal or experimental designs should be performed confirming current findings. Finally, we focused mainly on perceptions and attitudes toward different forms of VAW and gender-related individual and macrosocial factors separately. Therefore, based on a socio-ecological perspective of IPVAW [11], future research might allude to the interplay between different levels of social ecology to a better understand the implication of this social and health problem.

## 5. Conclusions 

As IPVAW victims disclose their victimization experiences more readily to informal support, public helping reactions to known IPVAW victims can represent an essential point to their recovery and commitment during the legal process. Examining the gender-related mechanisms that underlie helping reactions to IPVAW provided promising conclusions derived from this preliminary European community-sample study. As expected, at the individual level, Europeans who endorsed unsupportive attitudes and positive perceptions about different forms of violence against women, engaged with informal and formal helping reactions toward known IPVAW victims. At the country level, empirical evidence showed that some types of help are provided independently to a country’s gender-related ideological characteristics. Although formal reactions to known IPVAW victims have been found primarily within European countries with a greater perceived IPVAW prevalence and gender equality, informal reactions have been preferred in European countries with lesser perceived IPVAW prevalence and negative perceptions about the appropriateness of a legal response to psychological and sexual violence. Being aware of the time needed for changes to take effect, governments from the 28 European countries should start addressing policy efforts toward promoting country equality between women and men in domains such as knowledge, work, money, power, health, and time, as well as make all forms of violence against women equally visible and worrisome. In this vein, formal and informal helping reactions to IPVAW may join to guarantee victim safety.

## Figures and Tables

**Table 1:** Characteristics of the European population who knew a victim of IPVAW.

Sociodemographic Variables	Individual-Level Predictors	
	*n*	%
Gender		
Male	2455	34.5
Female	4660	65.5
Working Status		
Self-employed	496	7.0
Employed	3306	46.5
Not working	3313	46.6
Marital Status ^1^		
Unmarried	1225	17.2
Married/Single with partner	4464	62.7
Divorced or separated	755	10.6
Widowed	610	8.6
Years of education		
Up to 15 years	828	11.6
16–19 years	2992	42.1
20 years and older	2705	38.0
Still Studying	422	5.9
No full-time education	39	0.5
	M	SD
Age	48.83	17.07

Note: ^1^ The percentage of marital status does not add up to 100% because of missing data and “refusal” responses.

**Table 2:** Descriptive statistics for key predictor variables in the model at the individual and country levels.

**Individual-Level Predictors**					
**Attitudinal**	***N***	**M**	**SD**	**Min**	**Max**
Perceived prevalence of IPVAW	7115	1.87	0.66	1.00	4.00
Perceptions about the appropriateness of a legal response to psychological and sexual violence against partners	6790	1.94	0.54	1.00	4.00
VAW-supportive attitudes	5909	3.19	0.62	1.00	4.00
**Country-Level Predictors**					
**Ideological**		**M**	**SD**	**Min**	**Max**
Perceived prevalence of IPVAW	28	2.05	0.21	1.52	2.43
Perceptions about the appropriateness of a legal response to psychological and sexual violence against partners	28	1.99	0.14	1.74	2.37
VAW-supportive attitudes	28	3.06	0.26	2.58	3.60
**Structural**					
GEI Global	28	63.73	9.37	50.00	82.60

Note: IPVAW = intimate partner violence against women; VAW = violence against women; GEI = gender equality index.

**Table 3:** Odds ratio (95% confidence intervals [CI] from multilevel logistic regression models (random intercept and fixed slope) predicting formal helping reactions (FHRs), informal helping reactions (IHPs) and negative helping reactions (NHRs) to intimate partner violence against women in Europe (2016). Gender-related individual and sociodemographic predictors.

	Model 1			Model 2		
	FHR	IHR	NHR	FHR	IHR	NHR
	OR (CI)	OR (CI)	OR (CI)	OR (CI)	OR (CI)	OR (CI)
Fixed Effects						
Intercepts	0.11 *** (0.07–0.16)	1.25 (0.91–1.73)	0.64 ** (0.46–0.89)	0.10 *** (0.07–0.16)	1.53 * (1.07–2.18)	0.52 *** (0.36–0.74)
Male	Reference	Reference	Reference	Reference	Reference	Reference
Female	1.65 *** (1.43–1.92)	1.54 *** (1.38–1.71)	0.58 *** (0.52–0.65)	1.49 *** (1.26–1.76)	1.35 *** (1.20–1.54)	0.67 *** (0.59–0.77)
Self–employed	Reference	Reference	Reference	Reference	Reference	Reference
Employed	1.15 (0.87–1.52)	0.87 (0.71–1.08)	1.08 (0.86–1.34)	1.17 (0.86–1.58)	0.87 (0.69–1.10)	1.09 (0.85–1.39)
Not working	1.17 (0.88–1.57)	0.84 (0.67–1.04)	1.13 (0.90–1.42)	1.17 (0.85–1.61)	0.85 (0.66–1.08)	1.11 (0.85–1.43)
Unmarried	Reference	Reference	Reference	Reference	Reference	Reference
Married/Single with partner	0.86 (0.72–1.04)	1.04 (0.90–1.21)	1.00 (0.86–1.17)	0.90 (0.73–1.10)	1.04 (0.88–1.23)	1.01 (0.84–1.20)
Divorced or separated	1.33 * (1.04–1.70)	1.18 (0.96–1.47)	0.77 * (0.61–0.97)	1.27 (0.96–1.68)	1.10 (0.87–1.40)	0.81 (0.62–1.05)
Widowed	0.77 (0.56–1.05)	0.91 (0.72–1.16)	1.21 (0.94–1.56)	0.77 (0.54–1.10)	0.91 (0.68–1.20)	1.28 (0.96–1.72)
Up to 15 years	Reference	Reference	Reference	Reference	Reference	Reference
16–19 years	1.03 (0.83–1.28)	1.17 * (1.001–1.38)	0.85 (0.72–1.00)	1.12 (0.87–1.43)	1.12 (0.93–1.34)	0.89 (0.73–1.08)
20 years and older	1.24 (0.99–1.55)	1.35 *** (1.14–1.60)	0.71 * (0.60–0.85)	1.33 * (1.03–1.72)	1.25 * (1.02–1.52)	0.77 * (0.62–0.94)
Still Studying	0.66 (0.43–1.02)	1.00 (0.74–1.36)	1.09 (0.79–1.50)	0.79 (0.49–1.28)	0.97 (0.69–1.38)	1.14 (0.80–1.65)
No full–time education	2.79 ** (1.35–5.76)	0.87 (0.45–1.70)	0.96 (0.48–1.94)	2.93 * (1.18–7.29)	0.68 (0.29–1.55)	1.36 (0.58–3.21)
Age	1.00 (0.99–1.00)	0.99 * (0.99–0.999)	1.00 * (1.00–1.01)	1.00 (0.99–1.01)	1.00 (0.99–1.002)	1.00 (1.00–1.01)
Perceived prevalence of IPVAW (L–1)				0.70 *** (0.62–0.80)	0.89 * (0.81–0.98)	1.20 *** (1.10–1.32)
Perceptions about the appropriateness of a legal response to psychological and sexual violence against partners (L–1)				0.77 *** (0.67–0.90)	0.94 (0.84–1.05)	1.14 * (1.01–1.28)
VAW–supportive attitudes (L–1)				1.14 (1.00–1.31)	1.32 *** (1.19–1.46)	0.73 *** (0.66–0.81)
Random effects						
Between–country variance	0.16	0.20	0.18	0.14	0.22	0.18
AIC	6086.0	8880.2	8191.7	4887.1	6935.1	6330.8
BIC	6175.2	8969.4	8280.9	4993.2	7041.2	6436.9
Deviance	6060.0	8854.2	8165.7	4855.1	6903.1	6298.8
N (L–1)	7054	7054	7054	5612	5612	5612
N (L–2)	28	28	28	28	28	28

Note: *p*-values correspond to logit-odd estimates; IPVAW = intimate partner violence against women; VAW = violence against women; CI = confidence interval; FHR = formal helping reaction to IPVAW; IHP = informal helping reaction to IPVAW; and NHR = negative helping reaction to IPVAW; N (L−1) = total number of participants; N (L−2) = total number of countries; * *p* < 0.05. ** *p* < 0.01. *** *p* < 0.001.

**Table 4:** Odds ratio (95% confidence intervals) from multilevel logistic regression models (random intercept and fixed slope) predicting formal helping reactions (FHRs), informal helping reactions (IHPs), and negative helping reactions (NHRs) to intimate partner violence against women in Europe (2016). Sociodemographic and gender-related individual and macrosocial predictors.

	Model 3			Model 4		
	FHR	IHR	NHR	FHR	IHR	NHR
	OR (95% CI)	OR (95% CI)	OR (95% CI)	OR (95% CI)	OR (95% CI)	OR (95% CI)
Fixed effects						
Intercepts	0.10 *** (0.07–0.16)	1.57 ** (1.12–2.20)	0.50 *** (0.35–0.72)	0.10 *** (0.07–0.17)	1.57 ** (1.12– 2.20)	0.50 *** (0.35–0.71)
Male	Reference	Reference	Reference	Reference	Reference	Reference
Female	1.50 *** (1.26–1.77)	1.36 *** (1.20–1.54)	0.67 *** (0.59–0.77)	1.50 *** (1.27–1.78)	1.36 *** (1.20–1.54)	0.67 *** (0.59–0.77)
Self–employed	Reference	Reference	Reference	Reference	Reference	Reference
Employed	1.18 (0.87–1.60)	0.88 (0.69–1.11)	1.08 (0.84–1.38)	1.17 (0.86–1.58)	0.88 (0.70–1.11)	1.08 (0.84–1.38)
Not working	1.18 (0.86–1.62)	0.85 (0.67–1.09)	1.10 (0.85–1.42)	1.14 (0.83–1.57)	0.86 (0.67–1.10)	1.10 (0.85–1.42)
Unmarried	Reference	Reference	Reference	Reference	Reference	Reference
Married/Single with partner	0.90 (0.73–1.10)	1.04 (0.88–1.23)	1.00 (0.84–1.20)	0.88 (0.72–1.08)	1.04 (0.88–1.23)	1.01 (0.84–1.20)
Divorced or separated	1.28 (0.97–1.69)	1.11 (0.87–1.41)	0.81 (0.62–1.05)	1.28 (0.97–1.69)	1.11 (0.88–1.42)	0.80 (0.62–1.05)
Widowed	0.79 (0.55–1.13)	0.91 (0.69–1.20)	1.28 (0.95–1.71)	0.78 (0.54–1.11)	0.91 (0.69–1.21)	1.27 (0.95–1.71)
Up to 15 years	Reference	Reference	Reference	Reference	Reference	Reference
16–19 years	1.14 (0.89–1.47)	1.11 (0.92–1.33)	0.90 (0.74–1.09)	1.13 (0.88–1.46)	1.10 (0.91–1.32)	0.91 (0.75–1.10)
20 years and older	1.36 * (1.05–1.76)	1.23 * (1.01–1.50)	0.78 * (0.63–0.95)	1.30 * (1.003–1.68)	1.22 (0.99–1.49)	0.79 * (0.64–0.97)
Still Studying	0.81 (0.50–1.31)	0.96 (0.68–1.36)	1.16 (0.81–1.67)	0.80 (0.49–1.30)	0.94 (0.66–1.34)	1.18 (0.82–1.70)
No full–time education	2.85 * (1.15–7.08)	0.67 (0.29–1.55)	1.38 (0.58–3.24)	2.74 * (1.11–6.80)	0.68 (0.30–1.57)	1.36 (0.58–3.21)
Age	1.00 (0.99–1.01)	1.00 (0.99–1.001)	1.00 (0.99–1.01)	1.00 (0.99–1.01)	1.00 (0.99–1.001)	1.00 (0.99–1.01)
Perceived prevalence of IPVAW (L–1)	0.70 *** (0.62–0.80)	0.89 * (0.81–0.98)	1.20 *** (1.09–1.28)	0.71 *** (0.63–0.80)	0.89 * (0.81–0.98)	1.20 *** (1.09–1.32)
Perceptions about the appropriateness of a legal response to psychological and sexual violence against partners (L–1)	0.77 *** (0.67–0.90)	0.94 (0.84–1.05)	1.13 * (1.01–1.28)	0.77 *** (0.66–0.89)	0.94 (0.84–1.06)	1.13 * (1.003–1.27)
VAW–supportive attitudes (L–1)	1.14 (0.99–1.31)	1.32 *** (1.19–1.47)	0.73 *** (0.66–0.81)	1.15 * (1.001–1.32)	1.32 *** (1.19–1.46)	0.73 *** (0.66–0.81)
Interview privacy				1.21 * (1.04–1.40)	1.00 (0.88–1.13)	0.98 (0.86–1.11)
Respondent’s apparent interest during the interview				1.09 (0.95–1.25)	1.05 (0.95–1.17)	0.94 (0.85–1.05)
Perceived prevalence of IPVAW (L–2)	0.60 (0.33–1.10)	2.25 * (1.14–4.44)	0.53 (0.26–1.06)	0.46 ** (0.28–0.76)	2.35 * (1.16–4.74)	0.56 (0.27–1.15)
Perceptions about the appropriateness of a legal response to psychological and sexual violence against partners (L–2)	0.44 (0.15–1.30)	6.78 ** (2.00–23.0)	0.20 * (0.06–0.71)	0.73 (0.30–1.74)	6.37 ** (1.83–22.19)	0.18 * (0.05–0.65)
VAW–supportive attitudes (L–2)	1.68 (0.90–3.15)	3.23 ** (1.58–6.63)	0.31 ** (0.15–0.64)	0.72 (0.38–1.38)	3.71 ** (1.48–9.29)	0.37 * (0.14–0.95)
GEI				1.03 *** (1.02–1.06)	0.99 (0.97–1.02)	0.99 (0.97–1.02)
Random effects						
Between–country variance	0.08	0.13	0.14	0.03	0.13	0.14
AIC	4881.6	6928.0	6326.4	4872.5	6932.7	6330.6
BIC	5007.6	7054.1	6452.4	5051.6	7078.7	6476.5
Deviance	4843.6	6890.0	6288.4	4818.5	6888.7	6286.6
N (L–1)	5612	5612	5612	5612	5612	5612
N (L–2)	28	28	28	28	28	28

Note: *p*-values correspond to logit-odd estimates; IPVAW = intimate partner violence against women; VAW = violence against women; CI = confidence interval; FHR = formal helping Reaction to IPVAW; IHP = informal helping reaction to IPVAW; and NHR = negative helping reaction to IPVAW; N (L–1) = total number of participants; N (L–2) = total number of countries; * *p* < 0.05, ** *p* < 0.01, *** *p* < 0.001.

## Supplementary Materials

The following one is available online at https://www.mdpi.com/1660-4601/17/17/6314/s1, Table S1: Odds Ratio (95% confidence intervals) from Multilevel Logistic Regression Models (intercept-only model and random intercepts and fixed slopes model) predicting Formal Helping Reaction (FHR), Informal Helping Reaction (IHP) and Negative helping Reaction (NHR) to Intimate Partner Violence against Women in Europe (2016). Gender-related Individual and Country Predictors.

## References

[1] General Assembly of United Nations Declaration on the Elimination of Violence against Women. https://www.un.org/en/genocideprevention/documents/atrocity-crimes/Doc.21_declaration%20elimination%20vaw.pdf.

[2] World Health Organization, Pan American Health Organization (2012). Understanding and Addressing Violence against Women: Intimate Partner Violence.

[3] Zapata-Calvente A.L., Megías J.L., Moya M., Schoebi D. (2019). Gender-related ideological and structural macrosocial factors associated with intimate partner violence against European women. Psychol. Women Q..

[4] Gracia E., Martín-Fernández M., Lila M., Merlo J., Ivert A.K. (2019). Prevalence of intimate partner violence against women in Sweden and Spain: A psychometric study of the ‘Nordic paradox’. PLoS ONE.

[5] Ghose B., Yaya S. (2019). Experience of intimate partner violence and help-seeking behaviour among women in Uganda. Psych.

[6] World Bank Group (2020). Women, Business and the Law.

[7] Heise L.L. (2011). What Works to Prevent Partner Violence? An Evidence Overview.

[8] World Health Organization, London School of Hygiene and Tropical Medicine, South African Medinal Research Council (2013). Global and Regional Estimates of Violence against Women: Prevalence and Health Effects of Intimate Partner Violence and Non-Partner Sexual Violence.

[9] European Union Agency for Fundamental Rights (2014). Violence against Women: An EU-Wide Survey.

[10] Leone R.M., Parrott D.J., Swartout K.M. (2016). Masculinity and Bystander Attitudes: Moderating Effects of Masculine Gender Role Stress. Psychol. Violence.

[11] Heise L.L. (1998). Violence against women: An integrated, ecological framework. Violence Woman.

[12] Abramsky T., Watts C.H., Garcia-Moreno C., Devries K., Kiss L., Ellsberg M., Jansen H.A., Heise L. (2011). What factors are associated with recent intimate partner violence? Findings from the WHO multi-country study on women’s health and domestic violence. BMC Public Health.

[13] Heise L.L., Kotsadam A. (2015). Cross-national and multilevel correlates of partner violence: An analysis of data from population-based surveys. Lancet Glob. Health.

[14] Bograd M., Yllö K., Bograd B. (1998). Feminist perspectives on wife abuse: An introduction. Feminist Perspectives on Wife Abuse.

[15] Yodanis C.L. (2004). Gender inequality, violence against women, and fear: A cross-national test of the feminist theory of violence against women. J. Interpers. Violence.

[16] McCarthy K.J., Mehta R., Haberland N.A. (2018). Gender, power, and violence: A systematic review of measures and their association with male perpetration of IPV. PLoS ONE.

[17] Waltermaurer E. (2012). Public justification of intimate partner violence: A review of the literature. Trauma Violence Abuse.

[18] Ackerson L.K., Subramanian S.V. (2008). State gender inequality, socioeconomic status and intimate partner violence (IPV) in India: A multilevel analysis. Aust. J. Soc. Issues.

[19] Beyer K., Wallis A.B., Hamberger L.K. (2015). Neighborhood environment and intimate partner violence: A systematic review. Trauma Violence Abuse.

[20] Banyard V.L. (2011). Who will help prevent sexual violence: Creating an ecological model of bystander intervention. Psychol. Violence.

[21] Sylaska K.M., Edwards K.M. (2014). Disclosure of intimate partner violence to informal social support network members: A review of the literatura. Trauma Violence Abuse.

[22] Gracia E., Martín-Fernández M., Marco M., Santirso F.A., Vargas V., Lila M. (2018). The willingness to intervene in cases of intimate partner violence against women (WI-IPVAW) scale: Development and validation of the long and short versions. Front. Psychol..

[23] Latane B., Darley J.M. (1970). The Unresponsive Bystander: Why Doesn’t he Help?.

[24] Burn S. (2009). A situational model of sexual assault prevention through bystander intervention. Sex Roles.

[25] Berkowitz A. (2009). Response Ability: Complete Guide on Bystander Behavior.

[26] Flood M., Pease B. (2009). Factors influencing attitudes to violence against women. Trauma Violence Abuse.

[27] Baldry A.C., Pagliaro S. (2014). Helping victims of intimate partner violence: The influence of group norms among lay people and the police. Psychol. Violence.

[28] Cinquegrana V., Baldry A.C., Pagliaro S. (2018). Intimate partner violence and bystanders’ helping behaviour: An experimental study. J. Aggression Conflict Peace Res..

[29] Beeble M.L., Post L.A., Bybee D., Sullivan C.M. (2008). Factors related to willingness to help survivors of intimate partner violence. J. Interpers. Violence.

[30] Megías J.L., Toro-García V., Carretero-Dios H. (2018). The Acceptance of Myths About Intimate Partner Violence Against Women (AMIVAW) Scale: Development and Validation in Spanish and English. Psychol. Women Q..

[31] Yamawaki N., Ochoa-Shipp M., Pulsipher C., Harlos A., Swindler S. (2012). Perceptions of domestic violence: The effects of domestic violence, myth, victim’s relationship with her abuser, and the decision to return to her abuser. J. Interpers. Violence.

[32] Pagliaro S., Pacilli M.G., Baldry A.C. (2020). Bystanders’ reactions to intimate partner violence: An experimental approach. Eur. Rev. Soc. Psychol..

[33] Banyard V.L. (2015). Toward the Next Generation of Bystander Prevention of Sexual and Relationship Violence: Actions Coils to Engage Community.

[34] Hofstede G.H., Hofstede G.J., Minkov M. (2010). Cultures and Organizations: Software of the Mind.

[35] Ebbeler C., Grau I., Banse R. (2017). Cultural and individual factors determine physical aggression between married partners: Evidence from 34 countries. J. Cross Cult. Psychol..

[36] Mallory A.B., Dharnidharka P., Deitz S.L., Barros-Gomes P., Cafferky B., Stith S.M., Van K. (2016). A meta-analysis of cross cultural risk markers for intimate partner violence. Aggression Violent Behav..

[37] Nguyen T.T., Morinaga Y., Frieze I.H., Cheng J., Li M., Doi A., Hirai T., Joo E., Li C. (2013). College students’ perceptions of intimate partner violence: A comparative study of Japan, China, and the United States. Int. J. Confl. Violence.

[38] Heath R. (2012). Women’s Access to Labor Market Opportunities, Control of Household Resources, and Domestic Violence.

[39] Archer J. (2006). Cross-cultural differences in physical aggression between partners: A social-role analysis. Pers. Soc. Psychol. Rev..

[40] Banyard V.L., Moynihan M.M., Cares A.C., Warner R. (2014). How do we know if it works? Measuring outcomes in bystander-focused abuse prevention on campuses. Psychol. Violence.

[41] Stein J.L. (2007). Peer educators and close friends as predictors of male college students’ willingness to prevent rape. J. Coll. Stud. Dev..

[42] Jewkes R., Flood M., Lang J. (2015). From work with men and boys to changes of social norms and reduction of inequities in gender relations: A conceptual shift in prevention of violence against women and girls. Lancet.

[43] European Commission (2016). Special Eurobarometer 449: Gender-Based Violence.

[44] European Institute for Gender Equality (2017). Gender Equality Index, 2017. Measuring Gender Equality in the European Union 2005–2015.

[45] Banyard V.L. (2008). Measurement and correlates of pro-social bystander behavior: The case of interpersonal violence. Violence Vict..

[46] Herrero J., Rodriguez F.J., Torres A. (2017). Acceptability of partner violence in 51 societies: The role of sexism and attitudes toward violence in social relationships. Violence Women.

[47] Schafer J.L. (1999). Multiple imputation: A primer. Stat. Methods Med. Res..

[48] Bennett D.A. (2001). How can I deal with missing data in my study?. Aust. N. Z. J. Public Health.

[49] Merlo J., Chaix B., Yang M., Lynch J., Råstam L. (2005). A brief conceptual tutorial of multilevel analysis in social epidemiology: Linking the statistical concept of clustering to the idea of contextual phenomenon. J. Epidemiol. Community Health.

[50] Longford N.T. (1993). Regression analysis of multilevel data with measurement error. Br. J. Math. Stat. Psychol..

[51] Heck R., Thomas S.L. (2015). An Introduction to Multilevel Modeling Techniques: MLM and SEM Approaches Using Mplus.

[52] Hox J.J. (2010). Multilevel Analysis: Techniques and Aplications.

[53] Akinwande M.O., Dikko H.G., Samson A. (2015). Variance inflation factor: As a condition for the inclusion of suppressor variable(s) in regression analysis. Open J. Stat..

[54] Bates D., Maechler M., Bolker B.M., Walker S. (2015). Fitting linear mixed-effects models using lme4. J. Stat. Softw..

[55] LeBreton J.M., Senter J.L. (2008). Answers to 20 questions about interrater reliability and interrater agreement. Organ. Res. Methods.

[56] Bliese P.D. (1998). Group size, ICC valies, and group-level correlations: A simulation. Organ. Res. Methods.

[57] Merino J. (2017). The potential of multilevel logistic regression. A proposal for implementation in self-perceibed-health. EMPIRIA.

[58] Robinson S.R., Ravi K., Voth Schrag R.J. (2020). A Systematic review of barriers to formal help seeking for adult survivors of IPV in the United States, 2005–2019. Trauma Violence Abuse.

[59] Finnegan H.A., Timmons P.A. (2012). Differential effects of gender on perceptions of stalking and harassment behavior. Violence Vict..

[60] Banyard V.L., Moynihan M.M. (2011). Variation in bystander behavior related to sexual and intimate partner violence prevention: Correlates in a sample of college students. Psychol. Violence.

[61] Gracia E., Herrero J. (2006). Public attitudes toward reporting partner violence against women and reporting behavior. J. Marriage Fam..

[62] Wilson W.J. (2009). More Than Just Race.

[63] Carlson M. (2008). I’d rather go along and be considered a man: Masculinity and bystander intervention. J. Men’s Stud..

[64] Baldry A.C., Pacilli M.G., Pagliaro S. (2015). She’s not a person … she’s just a woman! Infra-humanization and intimate partner violence. J. Interpers. Violence.

[65] Waltermaurer E. (2007). Differentiating between intimate partner violence and stranger violence risk among women through an examination of residential change. Fem. Criminol..

[66] Frye V., Wilt S. (2001). Femicide and social disorganization. Violence Women.

[67] Gracia E., García F., Lila M. (2009). Public responses to intimate partner violence against women: The influence of perceived severity and personal responsibility. Span. J. Psychol..

[68] Klein E., Campbell J., Soler E., Ghez M. (1997). Ending Domestic Violence: Changing Public Perceptions/Halting the Epidemic.

[69] Yamawaki N., Ostenson J.A., Brown C.R. (2009). The functions of gender role traditionality, ambivalent sexism, injury, and frequency of assault on domestic violence perception: A study between Japanese and American college students. Violence Women.

[70] Fischer P., Krueger J.I., Greitemeyer T., Vogrincic C., Kastenmuller A., Frey D., Heene M., Wicher M., Kainbacher M. (2011). The bystander-effect: A meta-analytic review on bystander intervention in dangerous and non-dangerous emergencies. Psychol. Bull..

[71] Ermer A.E., Roach A.L., Coleman M., Ganong L. (2017). Deconstructing attitudes about intimate partner violence and bystander intervention: The roles of perpetrator gender and severity of aggression. J. Interpers. Violence.

[72] Postmus J.L., Hoge G.L., Breckenridge J., Sharp-Jeffs N., Chung D. (2020). Economic Abuse as an Invisible Form of Domestic Violence: A Multicountry Review. Trauma Violence Abuse.

[73] Levine M., Crowther S. (2008). The responsive bystander: How social group membership and group size can encourage as well as inhibit bystander intervention. J. Pers. Soc. Psychol..

[74] Bierbrauer G. (1994). Toward an understanding of legal culture: Variations in individualism and collectivism between Kurds, Lebanese, and Germans. Law Soc. Rev..

[75] Hayes B.E., Boyd K.A. (2017). Influence of individual- and national-level factors on attitudes toward intimate partner violence. Sociol. Perspect..

[76] Kelly J.A. (2004). Popular opinion leaders and HIV prevention peer education: Resolving discrepant findings, and implications for the development of effective community programmes. AIDS Care.

[77] Michau L., Horn J., Bank A., Dutt M., Zimmerman C. (2015). Prevention of violence against women and girls: Lessons from practice. Lancet.

[78] Breakthrough (2013). Breakthrough’s Bell Bajao! A Campaign to Bring Domestic Violence to a Halt.

[79] WLUML (2011). Strategies of Resistance: Challenging the Cultural Disempowerment of Women.

[80] Esplen E. (2006). Engaging Men in Gender Equality: Positive Strategies and Approaches.

[81] Michau L. (2007). Approaching old problems in new ways: Community mobilization as a primary prevention strategy to combat violence against women. Gend. Dev..

[82] Brenner A. (2013). Resisting simple dichotomies: Critiquing narratives of victims, perpetrators, and harm in feminist theories of rape. Harv. J. Law Gend..

[83] Miller V., VeneKlasen L., Reilly M., Clark C. (2006). Power: Concepts for Revisioning Power for Justice, Equality and Peace.

[84] Leung K., Van de Vijver F.J.R. (2008). Strategies for strengthening causal inferences in cross-cultural research: The consilience approach. Int. J. Cross Cult. Manag..

[85] Benítez I., Padilla J.L., Hidalgo M.D., Sireci S.G. (2016). Using mixed methods to interpret differential item functioning. J. Appl. Meas. Educ..

