# Supplementary material for: Public Helping Reactions to Intimate Partner Violence against Women in European Countries: The Role of Gender-Related Individual and Macrosocial Factors
Source: Int J Environ Res Public Health. 2020 Aug 30;17(17):6314. doi: 10.3390/ijerph17176314 (PMC7503533; doi:10.3390/ijerph17176314)
Supplement: Supplementary file 1 [file ijerph-17-06314-s001.pdf]

Table S1. Odds Ratio (95% confidence intervals) from Multilevel Logistic Regression Models (intercept-only model and random intercepts and fixed slopes model) predicting Formal Helping Reaction (FHR), Informal Helping Reaction (IHP) and Negative helping Reaction (NHR) to Intimate Partner Violence against Women in Europe (2016). Gender-related Individual and Country Predictors

	**Model 0**			**Model 4**		
	**FHR**	**IHR**	**NHR**	**FHR**	**IHR**	**NHR**
	**OR (95% IC)**	**OR (95% IC)**	**OR (95% IC)**	**OR (95% IC)**	**OR (95% IC)**	**OR (95% IC)**
Fixed effects						
Intercepts	0.18 (0.15-0.21)	1.76 (1.48-2.09)	0.41 (0.35-0.49)	0.11*** (0.07-0.16)	1.57** (1.12-2.19)	0.50*** (0.35-0.71)
Male				Reference	Reference	Reference
Female				1.50*** (1.27-1.78)	1.36*** (1.19-1.54)	0.67*** (0.59-0.76)
Self-employed				Reference	Reference	Reference
Employed				1.17 (0.86-1.58)	0.88 (0.69-1.11)	1.08 (0.85-1.38)
Not working				1.15 (0.84-1.58)	0.85 (0.67-1.09)	1.10 (0.85-1.43)
Unmarried				Reference	Reference	Reference
Married/Single with partner				0.90 (0.73-1.10)	1.04 (0.88-1.23)	1.00 (0.84-1.20)
Divorced or separated				1.27 (0.97-1.68)	1.11 (0.87-1.41)	0.81 (0.62-1.05)
Widowed				0.77 (0.54-1.11)	0.91 (0.69-1.20)	1.28 (0.95-1.71)
Up to 15 years				Reference	Reference	Reference
16-19 years				1.14 (0.89-1.46)	1.11 (0.92-1.33)	0.90 (0.74-1.09)
20 years and older				1.30* (1.01-1.69)	1.23* (1.01-1.50)	0.78* (0.63-0.96)
Still Studying				0.81 (0.50-1.31)	0.96 (0.68-1.36)	1.16 (0.81-1.67)
No full-time education				2.76* (1.11-6.86)	0.67 (0.29-1.55)	1.38 (0.58-3.24)
Age				1.00 (0.99-1.01)	1.00 (0.99-1.00)	1.00 (1.00-1.01)
Perceived prevalence of IPVAW (L-1)				0.71*** (0.63-0.80)	0.89* (0.81-0.98)	1.20*** (1.09-1.32)
Perceptions about the appropriate legal response to psychological and sexual violence against partners (L-1)				0.77*** (0.69-0.89)	0.94 (0.84-1.05)	1.13* (1.01-1.28)
Attitudes toward				1.14	1.32***	0.73***

violence against women (L-1)				(1.00-1.31)	(1.19-1.47)	(0.66-0.81)
Perceived prevalence of IPVAW (L-2)				0.46** (0.32-0.98)	2.34* (1.16-4.73)	0.56 (0.27-1.15)
Perceptions about the appropriate legal response to psychological and sexual violence against partners (L-2)				0.72 (0.37-2.17)	6.37** (1.83-22.1)	0.18** (0.05-0.66)
Attitudes toward VAW (L-2)				0.72 (0.38-1.42)	3.69** (1.48-9.24)	0.37* (0.15-0.96)
GEI (L-2)				1.04*** (1.02-1.06)	0.99 (0.97-1.02)	0.99 (0.97-1.02)
Random effects						
Between-country variance	0.17	0.19	0.17	0.03	0.13	0.14
AIC	6202	9050.9	8397.4	4869.9	6929.8	6327.9
BIC	6215.8	9064.7	8411.1	5002.6	7062.5	6460.6
Deviance	6198	9046.9	8393.4	4829.9	6889.8	6287.9
N (L1)	7115	7115	7115	5612	5612	5612
N (L2)	28	28	28	28	28	28

p-values correspond to logit-odd estimates (* p< 0.05, **p <0.01, ***p< 0.001); VAW= Violence against women; CI = confidence Interval; FHR = Formal Helping Reaction to IPVAW; IHP = Informal Helping Reaction to IPVAW; NHR = Negative helping Reaction to IPVAW; GEI = Gender equality index
